# The Degree of Permafrost Degradation Determines the Stability of Soil Organic Matter

**DOI:** 10.3390/molecules31173069

**Published:** 2026-08-31

**Authors:** Siyuan Zou, Xiangwen Wu, Shuying Zang

**Affiliations:** 1 Modern Educational Technology and Experiment Centre, Harbin Normal University, Harbin 150025, China; 2 Heilongjiang Province Key Laboratory of Geographical Environment Monitoring and Spatial Information Service in Cold Regions, Harbin Normal University, Harbin 150025, China; 3 Key Laboratory of Geographical Processes and Ecological Security of Changbai Mountains, Ministry of Education, Northeast Normal University, Changchun 130024, China; 4 Institute of Natural Resources and Ecology, Heilongjiang Academy of Sciences, Harbin 150040, China; 5 Huzhong Permafrost and Cold Environments Observation and Research Station of Heilongjiang Province, Huzhong District, Daxing’anling 165036, China

**Keywords:** soil organic matter, stable isotopes, permafrost region, temperature sensitivity, mineral protection

## Abstract

Global warming profoundly alters the stability of soil organic matter (SOM) in permafrost zones, yet the underlying mechanisms governing SOM stability in permafrost landscapes at different stages of degradation remain unclear. This study utilised soil samples from the 0–50 cm layer collected from both continuous and isolated patches permafrost zones in the Daxing’an Mountains for laboratory incubation experiments at constant temperatures of 5 and 15 °C. The results for SOC, Fe and Al oxide content, δ ^13^C and MBC indicate that SOM in isolated patchy permafrost possesses greater bioavailability and higher decomposability under warming, and carbon mineralization in shallow soils is more temperature-sensitive (*Q*_10_ = 4.24). After 89 days of incubation, the mineralisation rate in mineral-rich deep soil was only half that of organic-rich surface soil. Furthermore, as the ratio of Fe/Al oxides to organic carbon increased, the sensitivity of SOM decomposition to temperature decreased significantly. Overall, the stability of organic matter in permafrost is jointly regulated by the intrinsic molecular properties of the organic matter and the protective effect of minerals; furthermore, this protective effect can significantly delay the process by which peatlands in permafrost regions transition from carbon sinks to carbon sources.

## 1. Introduction

Over the past decade, permafrost temperatures in polar and high-altitude regions have increased by 0.29 ± 0.12 °C, causing widespread permafrost degradation [1]. Under extreme future climate scenarios, near-surface permafrost coverage is projected to decline by 14% by the end of this century, with approximately 25% of stored permafrost organic carbon vulnerable to decomposition. This potential carbon loss is 2–3 times greater than the current global estimate of soil mineralization-derived carbon emissions [2,3]. Northern peatlands represent a critical terrestrial carbon reservoir, storing roughly one-third of global terrestrial organic carbon stocks [4]. Notably, mineral-rich deep organic carbon reservoirs in northern permafrost zones are capable of inducing substantial carbon–climate feedback, rendering them a core focus of current permafrost carbon cycle research [5,6,7].

The degradation of permafrost accelerates the release of organic carbon from the shallow soil profile, but the mechanisms by which organic carbon in the deep soil profile responds to these drivers remain unclear [8,9]. It is generally believed that organic carbon in soils with high mineral content is less sensitive to rising temperatures due to its longer turnover time and strong physicochemical sequestration. However, the IPCC assessment notes that the response of deep and shallow soils to rising temperatures will tend to converge over the next century [10]. It is generally accepted that the stability of SOM stems from its complex chemical structure and low-energy state, which either hinders microbial mineralization [11,12,13] or is determined by the formation of aggregates and physical adsorption [14,15,16]. Furthermore, mineral–organic interactions can transfer dissolved organic matter (DOM) from the liquid phase to the solid phase, reducing its availability and thereby promoting the long-term stability of SOM [17]. For example, clay minerals can increase the rate of microbial deposition and preserve SOM within interlayers [18]. present, the relative importance of mineral variables, molecular characteristics, and climatic conditions for soil organic matter (SOM) stability remains unquantified, especially in permafrost regions [19,20]. This knowledge gap hinders accurate prediction of both the magnitude and direction of permafrost–carbon–climate feedback. Therefore, systematic studies are urgently required. Such studies should focus on how mineral protection and organic-carbon molecular properties regulate soil organic matter decomposition. These efforts will help evaluate the potential risk of soil organic carbon loss across permafrost zones [21].

Most warming-simulation experiments support the following conclusion. Minerals provide physical protection. This protection suppresses microbial mineralization of mineral associated organic carbon (MAOC) to different extents. Previous studies have found that global warming may significantly alter the stability of ‘old’ carbon in terrestrial soils [22], and that organic carbon in the lower layers of soil profiles, being protected by minerals, exhibits relatively low temperature sensitivity [23]. Structural equation modeling has further verified that mineral protection serves as a central regulator of soil organic carbon stability, sustaining long-term carbon preservation and attenuating the positive feedback between permafrost carbon decomposition and climate warming [24].

At present, research into the patterns of evolution in the stability of peatland soil organic matter driven by warming during permafrost degradation remains relatively scarce. To elucidate key scientific issues, such as the stability of soil organic matter and its sensitivity to temperature, in the context of permafrost degradation. Therefore, this study selected soil samples from the 0–50 cm soil layer of peatlands in both continuous and isolated patches of permafrost zones to conduct laboratory-scale simulated warming incubation experiments. In particular, the temperature sensitivity of soil carbon decomposition (usually defined as *Q*_10_, i.e., the factor by which the decomposition rate increases when the temperature rises by 10 °C) is determined by the chemical properties of the soil carbon itself and is used to quantify the stability of soil organic matter [25].

## 2. Materials and Methods

### 2.1. Study Site

The study site is situated in the Daxing’an Mountains (52°20′ N, 124°42′ E; Figure S1) and spans a south-to-north gradient, where permafrost distribution shifts from isolated patchy permafrost to discontinuous island-like thawed zones and finally to extensive continuous permafrost [26]. This region features a typical temperate continental climate with warm and humid summers, long and cold winters, a mean annual temperature of 2 °C, average annual precipitation of 430–530 mm, average annual relative humidity of 60–65 per cent, a frost-free period lasting 80–110 days, and pronounced spatial heterogeneity in local climate conditions. All sampling sites are covered by peat soil and permafrost, with consistent vegetation communities across the study area (Table S1). The shrub layer is predominated by *Ledum palustre*, *Rhododendron dauricum*, and *Vaccinium uliginosum*, while the herb layer is dominated by *Eriophorum vaginatum*. The study sites encompass two types of permafrost peatlands: continuous permafrost peatland (TQ) and isolated patchy permafrost peatland (JGDQ).

After field sampling, plant roots, sand, gravel, and other impurities were manually eliminated from the soil samples. The samples were stratified into five vertical layers within the 0–50 cm soil profile, namely 0–10 cm, 10–20 cm, 20–30 cm, 30–40 cm, and 40–50 cm. All samples were subsequently transported to the laboratory in insulated containers and preserved at 4 °C for subsequent analysis.

### 2.2. Sample Preparation

Soil samples were measured for soil organic carbon (SOC), dissolved organic carbon (DOC), and total nitrogen (TN). A stable isotope mass spectrometer (Mat253, Thermo Fisher Scientific, Waltham, MA, USA) was employed to determine the soil ^13^C isotopic composition before and after cultivation (SI). Soil texture was characterized via a laser particle size analyzer (Mastersizer 3000, Malvern, UK). Briefly, 2 g of air-dried soil samples sieved through a 2 mm mesh were pretreated with hydrogen peroxide to remove organic matter, with sodium hexametaphosphate applied as a stabilizing agent. Selective extraction procedures were performed to quantify the contents of free Fe/Al oxides (Fe_d_, Al_d_), poorly crystalline Fe/Al oxyhydroxides (Fe_o_, Al_o_), and organically complexed Fe/Al oxides (Fe_p_, Al_p_) in the soils (SI).

Two-level temperature warming incubation experiments were conducted in this study, with temperature settings of 15 °C and 5 °C (SI). Given the core focus on the impacts of varying warming magnitudes in the present study, shifts in bacterial community characteristics were considered temperature-driven variations and thus not further explored [27]. Gas concentrations were determined and analyzed using a gas chromatograph (Agilent 7890B GC System, Agilent Technologies, Santa Clara, CA, USA). The soil organic carbon mineralization rate in this study was defined as the CO_2_ production rate during soil incubation. Gas sampling was performed on days 1, 5, 11, 15, 21, 30, 42, 54, 61, 68, 75, and 89 throughout the entire incubation period. In total, 792 gas samples were measured, with the sample quantity calculated as: 2 temperature treatments × (30 soil samples + 3 blank controls) × 12 sampling replicates. The rate of soil organic carbon mineralisation was calculated as the difference between the CO_2_ concentration after incubation and that of the blank sample [28]. The cumulative amount of soil organic carbon mineralised at the end of the incubation period and the temperature sensitivity of soil organic carbon mineralisation were calculated using the equal-time method [29,30] (abbreviations in the formula are defined in the Supplementary Information), as follows:

$$F=\rho \times \Delta C\times V\times \frac{273}{\left(273+T\right)\times W}$$

$$C=\sum_{i=n}^{n}\frac{F_{i}+F_{i+1}}{2\times \left(t_{i+1}-t_{i}\right)\times 24}$$

$$Q_{10}={\left(\frac{K_{1}}{K_{2}}\right)}^{\frac{10}{T_{2}-T_{1}}}$$

Following an 89-day incubation experiments, soil DOC concentrations under different temperature treatments were measured, and infrared spectra of freeze-dried dissolved organic matter (DOM) were acquired. Fourier-transform infrared (FT-IR) spectroscopy was performed using a Vertex-80 spectrometer (Bruker, Billerica, MA, USA) with a KBr matrix, with a scanning wavenumber range of 400–4000 cm^−1^ [31]. For FT-IR sample preparation, 1 mg of freeze-dried DOM powder (previously oven-dried at a constant temperature of 65 °C for 24 h) was fully mixed with 100 mg of KBr and finely ground for 10 min before tableting [32]. Microbial carbon measurements were performed on the soil prior to cultivation (SI).

### 2.3. Statistical Analyses

All statistical analyses and figures were produced using Origin (2021). To determine the effects of temperature and composition, a one-way analysis of variance (ANOVA) was performed on the cumulative CO_2_-C emissions and DOC content of the two permafrost samples. Two-way ANOVA was conducted to evaluate the main and interactive effects of permafrost type and temperature on soil δ^13^C signatures, SOC mineralization properties, and functional component parameters (rA1637 and rA2926). Spearman’s correlation analysis was finally adopted to quantify the relationships among soil physicochemical indicators (SOC, initial δ^13^C values, Fe/Al oxides), organic carbon mineralization rates and temperature sensitivity, revealing the statistical mechanisms governing SOM stability responses to permafrost degradation and warming.

## 3. Results

### 3.1. Physico-Chemical Properties of Soil

The initial soil physicochemical properties of the 0–50 cm soil layer for the two permafrost peatland types are summarized in Table 1. For the TQ site, silt particles (2–20 μm) constituted 40.86–55.59% of the soil mass, sand particles (20–200 μm) accounted for 35.35–58.68%, and clay content increased vertically with soil depth, ranging from 0.45% to 10.29% (Table 1). For the JGDQ site, gravel content dominated the soil texture at 37.03–78.54%, while silt content increased markedly in the 20–50 cm soil layer.

At the TQ site, SOC and TN contents varied from 88.17 to 26.48 g/kg and 9.96 to 1.97 mg/g, respectively. At the JGDQ site, SOC content ranged from 348.40 to 55.12 g/kg, and TN content ranged from 42.43 to 5.48 mg/g. In general, both SOC and TN contents declined consistently with increasing soil depth across all sampling sites. Additionally, for both permafrost types, the TN concentration in the 0–10 cm topsoil was approximately 5–8 times that in the 40–50 cm subsoil, and deeper soil layers exhibited higher C:N ratios.

Across the two permafrost sites, total Fe and Al contents increased consistently with soil depth, with generally higher values observed in TQ than in JGDQ (Table 2). In terms of magnitude, the concentrations of different Fe and Al oxide forms followed a descending order: Fe_d_ > Fe_o_ > Al_d_ > Fe_p_ > Al_o_ > Al_p_. In both study areas, δ ^13^C values became progressively positive downward along the soil profile, which aligns with the typical isotopic fractionation pattern during soil organic matter decomposition. Notably, the TQ site exhibited higher overall δ ^13^C values than the JGDQ site. Furthermore, SOC concentrations were negatively correlated with δ ^13^C values and significantly positively correlated with the contents of organically complexed Fe/Al oxides (Table 3). 

### 3.2. Characteristics and Temperature Sensitivity of SOC Minerals

**Figure 1:**
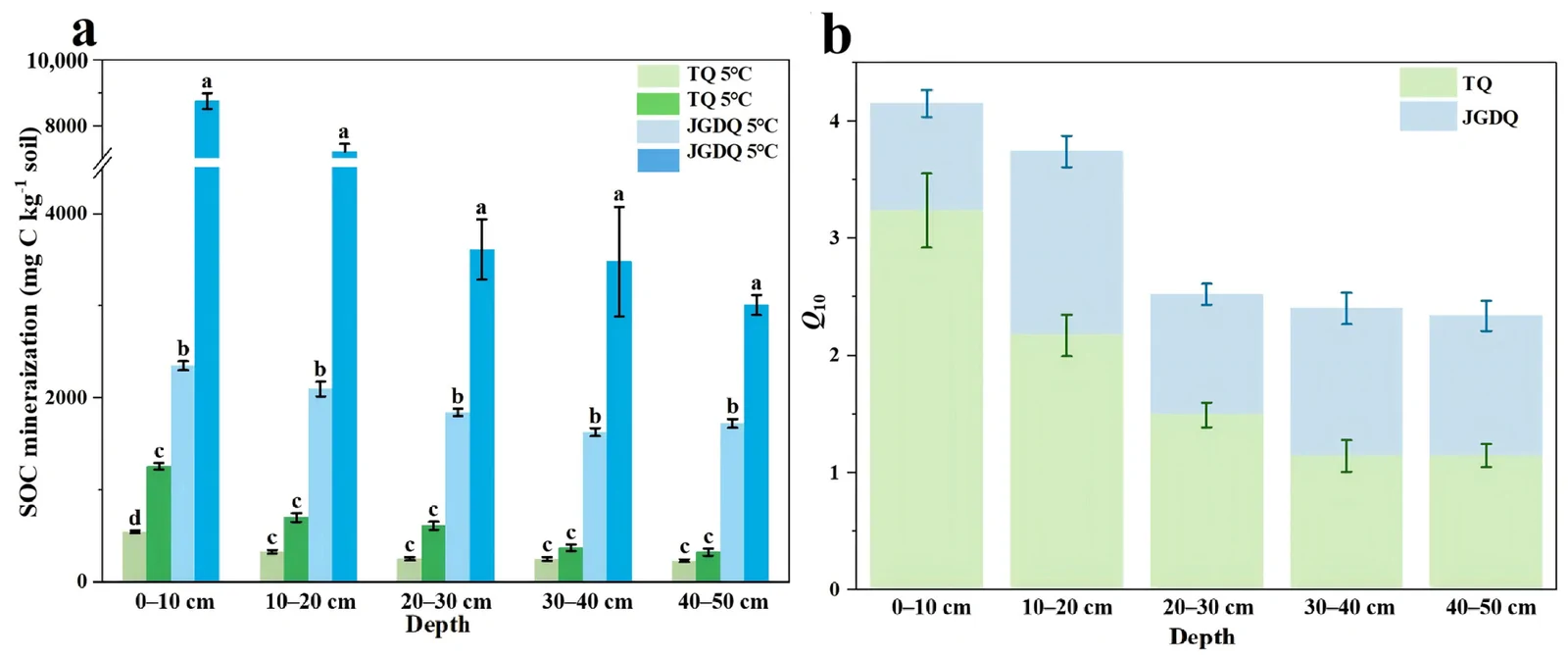
Organic carbon (OC) mineralization in soil samples from the continuous permafrost zone (TQ) and isolated patches permafrost zone (JGDQ) at different temperatures and depths after 89 days of cultivation ((**a**) cumulative CO_2_ emissions after 89-day incubation) and temperature sensitivity ((**b**) *Q*_10_). Significant differences between different types and soil depths are denoted by different letters (*p* < 0.05).

This study conducted an 89-day laboratory SOC mineralization incubation experiment to explore the temperature sensitivity of SOC mineralization in two distinct permafrost peatlands. At 5 °C, the cumulative SOC mineralization rates of the TQ site ranged from 227.33 to 538.20 mg C kg^−1^ OC^−1^, while those of the JGDQ site were 1719.07–2346.21 mg C kg^−1^. At 15 °C, the cumulative SOC mineralization values varied from 320.23 to 1249.70 mg C kg^−1^ OC^−1^ for TQ and 3007.84 to 8753.28 mg C kg^−1^ for JGDQ. Overall, the SOC mineralization flux was significantly greater in isolated patchy permafrost (JGDQ) than in continuous permafrost (TQ) (Figure 1a). After incubation, the magnitude of SOC mineralization increased by 1.41–1.75 times in deep soil layers and 2.32–3.73 times in shallow soil layers. Notably, SOC in the 10–50 cm soil profile of continuous permafrost showed an insignificant response to experimental warming.

The temperature sensitivity (*Q*_10_) of SOC mineralization exhibited a vertically decreasing trend along the soil profile in both permafrost peatland types, varying from 4.15 ± 0.11 to 1.14 ± 0.09. Overall, the TQ site presented lower *Q*_10_ values than the JGDQ site (Figure 1b). Correlation analysis revealed that *Q*_10_ was negatively associated with δ ^13^C values, metal-to-carbon ratios, clay content, and silt content, while being positively correlated with gravel content (Figure S4).

**Figure 2:**
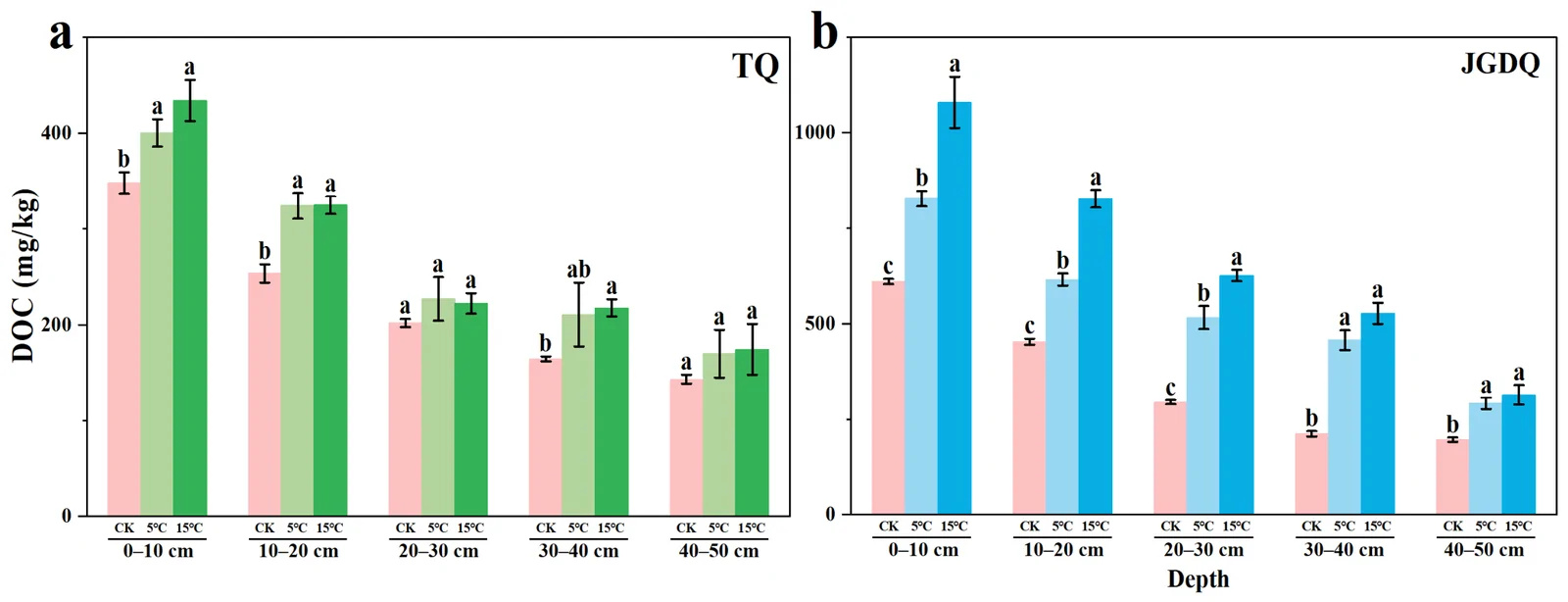
Soil dissolved organic carbon content before and after 0–50 cm soil cultivation in different types of permafrost zones ((**a**) continuous permafrost zone; (**b**) isolated patches permafrost zone). Significant differences between temperature and soil depth are indicated by different letters (*p* < 0.05).

With regard to DOC, the concentrations in the control groups of both permafrost types showed a decreasing trend at depths of 0–50 cm, following mineralization; DOC concentrations increased in all soil layers. Following warming incubation, deep soil DOC concentrations in the TQ site showed no significant response to temperature changes, whereas warming substantially altered DOC concentrations in the 0–30 cm soil layer of the JGDQ site (Figure 2).

Soil microbial biomass carbon (MBC) represents a labile organic carbon pool that participates in the short-term turnover of soil organic matter (SOM) and acts as a critical indicator of soil microbial carbon storage. The two permafrost types exhibited distinct vertical distribution patterns of MBC, with MBC concentrations declining consistently with increasing soil depth. Overall, the continuous permafrost site (TQ) had lower MBC contents than the isolated patchy permafrost site (JGDQ) (Figure S2).

### 3.3. ^13^C Isotopic and Molecular Characteristics

**Figure 3:**
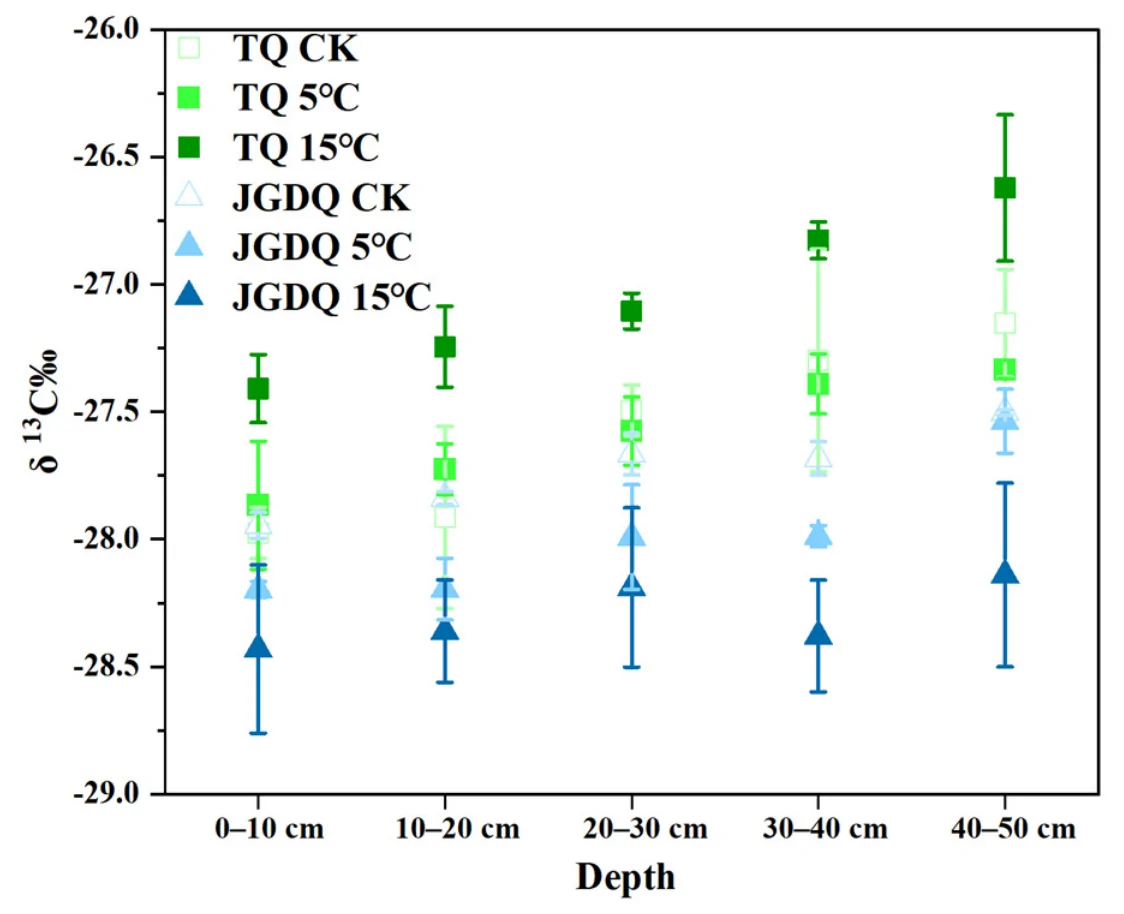
The δ ^13^C values of SOM in the control group at 0–50 cm depth across different types of permafrost zones under various incubation temperatures.

Analysis of variance revealed that experimental warming significantly altered soil organic matter (SOM) δ ^13^C values (Table 4). For the TQ site, δ ^13^C values increased after SOC mineralization at 5 °C, and the magnitude of such δ ^13^C enrichment was further enhanced at 15 °C. Moreover, the temperature-induced variations in δ ^13^C were more prominent in the 30–50 cm deep soil layer, while the 0–30 cm shallow layer exhibited relatively weaker temperature responses (Figure 3). In contrast, warming led to an overall reduction in soil δ ^13^C values at the JGDQ site. Collectively, SOM δ ^13^C values under warming conditions were lower in JGDQ than in TQ.

Table 4 presents significant interactive effects between permafrost type and temperature on key biogeochemical indicators in alpine peatland ecosystems. Permafrost type and temperature independently and jointly regulated soil δ ^13^C values and CO_2_ accumulation, with a significant interactive effect detected between the two factors. By contrast, permafrost type and temperature exhibited opposing influences on the spectral indices rA1367 and rA2926, while their interaction showed no significant effect on these two indicators.

**Figure 4:**
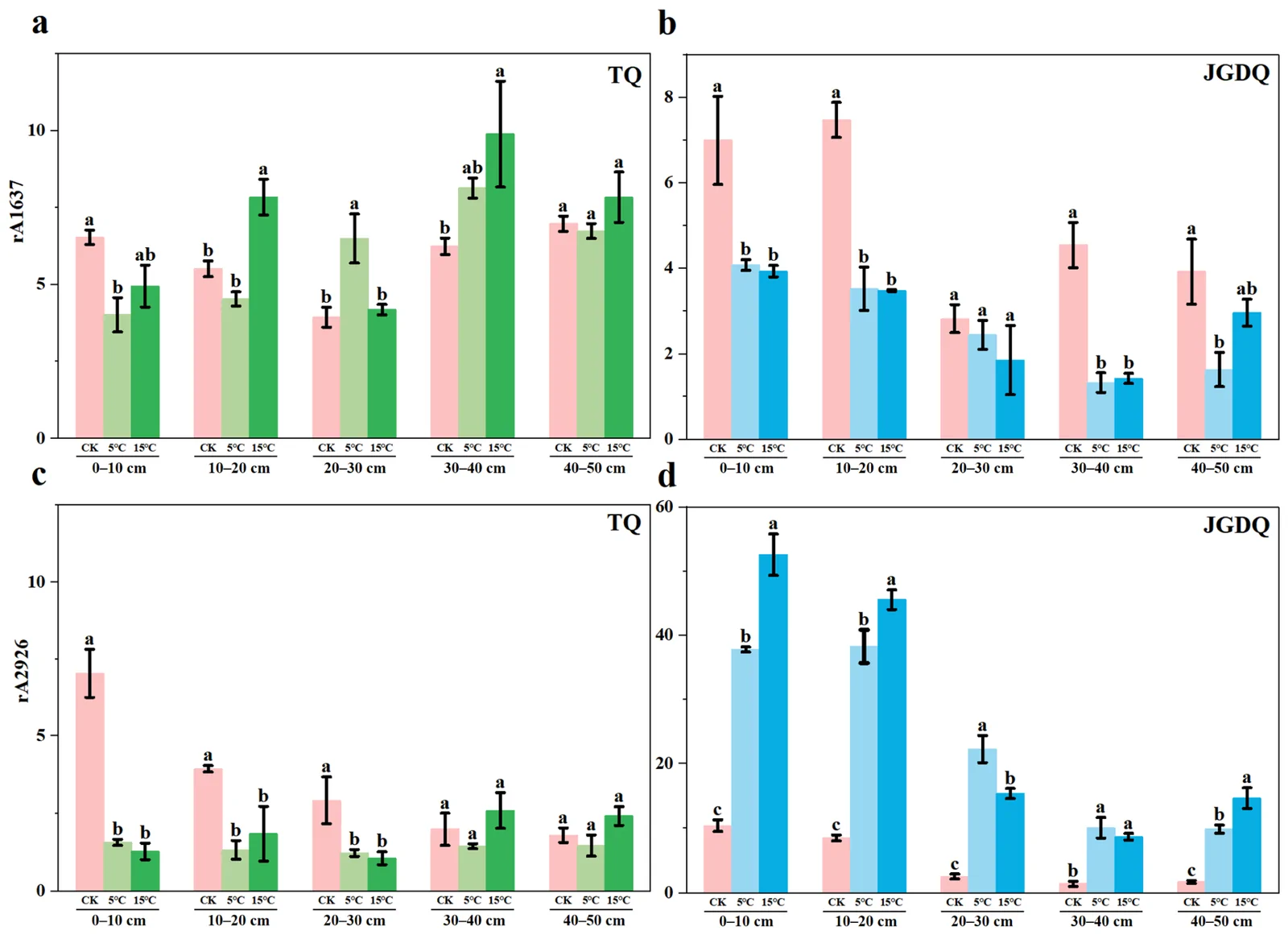
Values of rA1637 and rA2926 in the 0–50 cm soil layer in the continuous permafrost zone (TQ) and isolated patches permafrost zone (JGDQ) at different temperatures before and after 89 days of mineralization culture. Significant differences between temperature and soil depth are indicated by different letters (*p* < 0.05). (**a**) continuous permafrost (TQ), rA1637; (**b**) isolated patchy permafrost (JGDQ), rA1637; (**c**) continuous permafrost (TQ), rA2926; (**d**) isolated patchy permafrost (JGDQ), rA2926.

After incubation at 5 °C and 15 °C, distinct variations in spectral indices were observed between the two permafrost types (Figure S3). For continuous permafrost (TQ), the magnitude of the decline in rA2926 diminished with rising temperature, and rA2926 in the 30–50 cm soil layer remained insensitive to temperature elevation (Figure 4c). For isolated patchy permafrost (JGDQ), rA1637 in the 0–10 cm topsoil decreased significantly relative to the control group, while no significant differences in rA1637 were detected between the two warming treatments (Figure 4b). Overall, experimental warming decreased the relative proportion of labile organic matter in continuous permafrost but increased it in isolated patchy permafrost.

Comparisons of soil spectral indices between the two permafrost types after mineralization at two temperature treatments (Figure 4) indicated that for the continuous permafrost site (TQ), the stable spectral index rA1637 declined significantly in the 0–10 cm layer but rose substantially in the 20–50 cm layer. The labile spectral index rA2926 exhibited an obvious reduction after incubation, with the exception of the 30–40 cm layer. For the isolated patchy permafrost site (JGDQ), bulk SOM rA1637 values declined across the entire soil profile. Warming induced a prominent rise in rA2926 throughout the 0–50 cm soil layer, and such rA2926 values differed significantly between the two temperature gradients.

## 4. Discussion

### 4.1. Properties of SOC in Different Types of Permafrost Zones

Prior research reported that roughly 15% of soil organic carbon in alpine grasslands of the Tibetan Plateau is bound to iron, which corroborates the critical function of Fe and Al minerals in protecting organic carbon against decomposition within the permafrost region [33]. In the permafrost regions of the Daxing’an Mountains, SOC concentration has been found to correlate positively with free and organically complexed Fe/Al oxides, whilst showing a significant negative correlation with δ ^13^C values over many years; this is consistent with the findings of the present study, highlighting the important role of soil mineral phases in the formation and preservation of organic carbon (Table 3) [34]. In addition, total Fe and Al accumulated preferentially in deep soil horizons, further verifying that reactive minerals at depth facilitate long-term organic carbon persistence. Low-temperature-driven soil disturbances transport fresh soil organic matter (including DOC) downwards to deep layers; therein, DOC molecules bind to mineral surfaces via reactive hydroxyl (OH^−^) groups, and strong mineral adsorption inhibits microbial decomposition of these carbon pools [35,36].

In forest and grassland ecosystems, the δ^13^C values of organic carbon generally increase with increasing decomposition rate and soil depth, whereas SOC content and the C:N ratio decrease accordingly [37,38]. In the present study, shallow soils exhibited low δ^13^C values, and C:N ratios showed a positive linear relationship with δ ^13^C values-a trend inconsistent with universal patterns reported for organic soils. This discrepancy likely arises because deep-layer SOC is largely mineral-protected and therefore less susceptible to decomposition, resulting in more severe nitrogen loss relative to carbon loss. By comparison, prior research reported that δ ^13^C is positively correlated with C:N ratios in permafrost peatlands, as soil decomposition shifts from aerobic to anaerobic within the profile, δ ^13^C values first increase and then decrease with depth [38]. This study found similar results in isolated patches of permafrost. Furthermore, the δ ^13^C values in soils across the isolated patches permafrost zones were lower than those in soils beneath continuous permafrost areas, indicating that the decomposition process of SOC in continuous permafrost areas is weaker than in isolated patches permafrost zones, while also suggesting that minerals have an inhibitory effect on carbon mineralization.

### 4.2. The Effect of Warming on the Stability of SOM

Studies have shown that warming increases soil respiration rates, but the extent to which soil mineralization responds to temperature is determined by soil texture and biotic and abiotic properties [39,40]. In this study, permafrost type and temperature both had a significant influence on mineralization rates throughout the soil profile. The cumulative SOC mineralization in soils of isolated patches of permafrost was significantly greater than in continuous permafrost areas, indicating that soil organic matter in isolated patches of permafrost has higher potential availability (Figure 1a), whereas mineral-rich deep soils responded less strongly to warming than shallow soils with higher organic matter content. The type of permafrost has a significant effect on SOC mineralization in the shallow soil profile, whereas the warming effect on mineralization is not significant. Therefore, in the deep layers of peatlands within continuous permafrost zones, soil properties have a greater influence on SOC mineralization than temperature. These results suggest that, under warming conditions, higher mineral content in deep soils of continuous permafrost zones can mitigate the rate of soil organic carbon mineralization. However, the outlook for isolated patches of permafrost is not as optimistic.

Recalcitrant, aged soil organic carbon decomposes slowly, while labile organic carbon acts as the dominant substrate for microbial metabolism [37]. The mineralization of labile organic fractions also exhibits stronger temperature sensitivity than persistent organic carbon pools [41]. In our experiment, the relatively stable SOM fraction from continuous permafrost experienced greater decomposition within shallow soil layers. In contrast, the labile SOM fraction of isolated patchy permafrost increased significantly in the shallow layer (Figure 4). Long-term field observations have reported substantial accumulation of stable organic carbon in deep permafrost horizons, which can be attributed to enhanced mineral-associated protection. Meanwhile, SOM δ ^13^C values shift toward positive values under elevated temperatures, a signal that warming promotes the accumulation of stable SOC pools [21]. Our incubation results revealed elevated DOC concentrations across both permafrost types after warming treatments (Figure 2), which signals intensified SOM decomposition under thermal stimulation. Nevertheless, deep soils displayed milder DOC increments, demonstrating that mineral phases constrain the rate of SOM mineralization. Importantly, warming induced far larger DOC gains in JGDQ than in TQ. Since DOC is preferentially accessible to soil microbes, the organic carbon pool of isolated patchy permafrost will face greater decomposition risks under climate warming.

**Figure 5:**
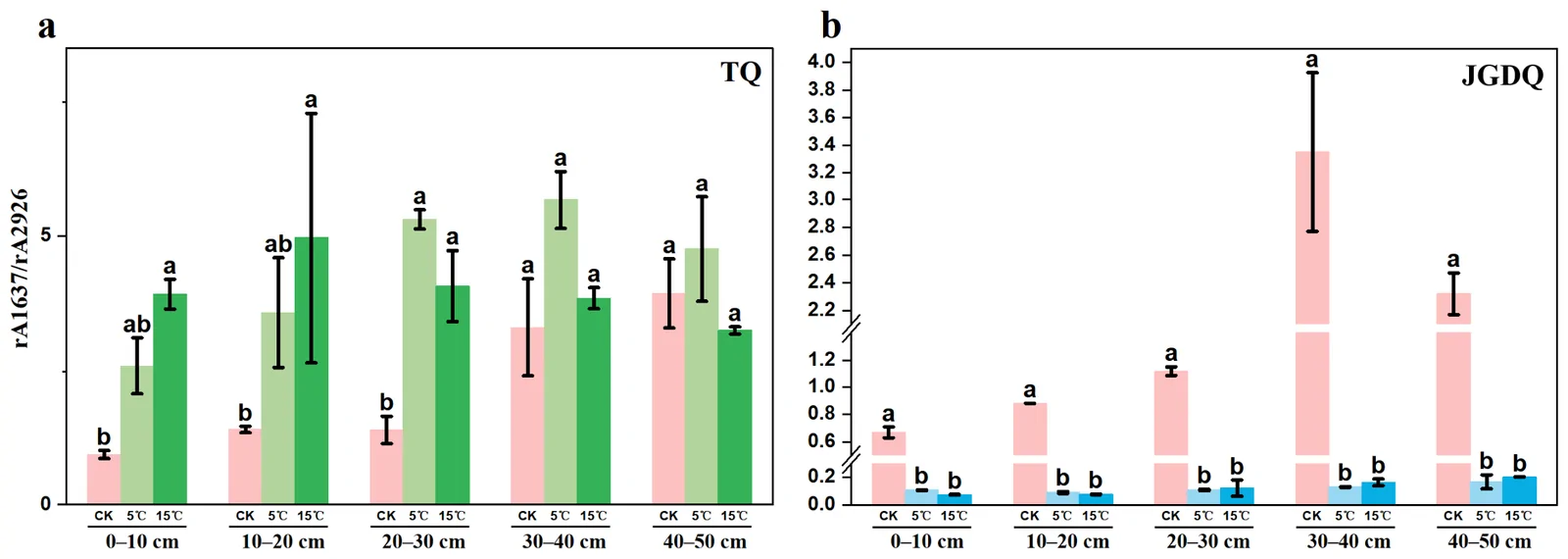
Values of rA1637/rA2926 (index of recalcitrance) [43] in the 0–50 cm soil in the continuous permafrost zone ((**a**) TQ) and isolated patches permafrost zone ((**b**) JGDQ) at different temperatures before and after 89 days of mineralization culture. Significant differences between temperature and soil depth are indicated by different letters (*p* < 0.05).

Decomposition of stable SOM fractions in permafrost soils has long been restrained under cold conditions. As illustrated in Figure 4a, the peak area of the stable spectral rA1637 declined within the 0–10 cm topsoil layer. Experimental warming mainly drove the decomposition of labile SOM fractions (Figure 4c), yet this thermal decomposition effect weakened substantially with increasing soil depth. Metal and metal oxide concentrations accumulated down the soil profile, while labile SOM exhibited no obvious depletion at depth. This evidence confirms that metal (oxyhydr) oxides exert a vital control over SOM persistence. Within the 30–50 cm soil layer, stable SOM fractions tended to rise under elevated temperatures, yet such temperature-induced increments lacked statistical significance. This reveals that temperature exerts a comparatively weak influence on the stability of deep SOM in continuous permafrost. Overall, warming strengthened SOM stability across the TQ soil profile. In contrast, in isolated patchy permafrost (JGDQ), lower Fe_p_ concentrations and weaker mineral protection mean that, under warming conditions, the stable SOM fraction (rA1637) becomes the primary substrate for decomposition (Figure 4b), as the reduced mineral protection fails to effectively shield these refractory components from microbial degradation [42]. This mechanistic explanation is further supported by the following observations: Fe_p_ oxides form stronger organo-mineral complexes than other types of metal oxides and thus provide the most significant protective effect, this also explains why the 30–40 cm soil layer, which has the lowest Fe_p_ content, exhibits the most pronounced decomposition of stable SOM (Figure 4b). After warming incubation, labile SOM rose significantly in the shallow horizons of isolated patchy permafrost (Figure 4d). This trend reveals that thermal elevation renders SOM more susceptible to decomposition and highlights the higher temperature sensitivity of SOM in JGDQ. By comparison, SOM in continuous permafrost maintained stronger stability under warming; mineral phases effectively shielded readily decomposable SOM against microbial breakdown. This mineral protection mechanism further accounts for the lower SOC mineralization magnitude observed in continuous permafrost relative to isolated patchy permafrost. Collectively, warming elevated overall SOM stability in continuous permafrost but induced a significant decline in SOM stability across isolated patchy permafrost (Figure 5).

Meanwhile, MOC is the primary organic carbon component in permafrost zones [44]. Mineral protection and microbial characteristics jointly determine the formation and stability of mineral-bound organic matter [42]. However, In the study area, microbial biomass carbon concentrations showed minimal variations in deep soil horizons (Figure S2), which partly accounts for the suppressed SOC mineralization rates at depth. The divergent decomposition patterns of SOM fractions between the two permafrost types can be mechanistically explained by mineral protection intensity and substrate accessibility. In continuous permafrost (TQ), higher mineral content (particularly Fe/Al oxides) provides stronger physicochemical protection, rendering mineral-associated organic matter less accessible to microbial decomposers. Under warming conditions, microorganisms preferentially utilize the more accessible labile SOM fractions (as indicated by declining rA2926), while stable fractions remain largely protected by mineral associations. In future studies on organic carbon decomposition in permafrost peatlands, particular attention should be paid to investigating the inhibitory effect of soil metal oxides on organic carbon mineralization and their coupling with microorganisms.

### 4.3. The Temperature Sensitivity of Soil Organic Matter and Mineral Protection

Complex organic molecules generally decompose slowly and have high activation energy thresholds. As soil organic matter rises in molecular weight and structural complexity, the energy input needed to fuel its biochemical turnover increases correspondingly. This translates to elevated activation energy and diminished thermal sensitivity of decomposition [25,45]. Our incubation experiment yielded higher *Q*_10_ values for isolated patchy permafrost relative to continuous permafrost (Figure 1b), a pattern driven by the abundant complex carbon compounds accumulated in JGDQ soils. From the perspective of soil texture, gravel-rich soils display stronger temperature sensitivity than clay-dominated soils [46]. Gravel-containing soils contain fine mineral fractions with large specific surface areas that readily bind with organic carbon, granting these particles strong adsorption and carbon preservation capacity [47]. The positive correlation between gravel proportion and thermal sensitivity detected in our study (Figure S4) aligns well with the carbon-temperature sensitivity framework.

Scanning electron microscopy (SEM) imaging demonstrated that after incubation at 5 °C, native cotton-like SOM evolved into fibrous structures (Figure S5). Incubation at 15 °C further intensified SOM fragmentation, forming flaky organic aggregates. Such drastic structural shifts illustrate that surface SOM (0–10 cm) in isolated patchy permafrost exhibits strong thermal sensitivity. Deep horizons containing structurally complex SOM displayed weaker temperature responses relative to topsoil, consistent with prior research conclusions [48]. Meanwhile, no obvious divergent structural variations in SOM were detected between the two temperature treatments (Figure S5c,d,g,h). Existing literature has reported that elevated gravel fractions in deep soils restrict the migration and interaction of microbes and extracellular enzymes. In our dataset, deep soils from continuous permafrost contained moderately higher gravel contents, which supports the above mechanism. Collectively, mineral-associated organic carbon protection coupled with suppressed microbial mobility jointly lowers the thermal sensitivity of SOM decomposition [49,50,51]. In addition, recent research reported minor vertical variations in *Q*_10_ values along soil profiles, whereas physicochemical mineral protection drives spatial heterogeneity in thermal sensitivity [48]. A higher metal: SOC ratio facilitates the combination between SOM and metal (oxyhydr)oxides, which strengthens mineral–carbon association [52,53]. Our results revealed a significant negative correlation between *Q*_10_ and the Fe/Al oxide-to-OC ratio (Figure S4). This relationship demonstrates that minerals with lower organic carbon loading possess greater adsorption affinity and exert more powerful protective effects on SOM. This mechanism further accounts for the minimum cumulative SOC mineralization detected in the 40–50 cm layer of continuous permafrost (Figure 1a): deep horizons accumulate the highest concentrations of Fe and Al oxides, and mineral-associated protection substantially suppresses organic carbon decomposition. The weak negative correlation between *Q*_10_ and δ ^13^C values further implies that minerals sequester partial labile SOM into stable organo-mineral complexes, which restrains SOC mineralization and lowers the thermal sensitivity of decomposition.

Organic compounds readily adsorb firmly onto precipitated Fe and Al oxide surfaces and associate with bulk SOM or crosslink with multifunctional organic moieties to form stabilizing agents. Such interactions facilitate particle aggregation, generate organo-mineral associations, and strengthen the structural stability of soil aggregates [54]. Among all measured indicators, crystalline Fe and amorphous Al oxides (Fe_d_/Al_d_) exhibited the tightest correlation with thermal sensitivity, which aligns with their inherent properties and validates the mineral-driven stabilization pathway for SOM in permafrost peatlands. Parallel investigations have identified identical mineral protection mechanisms for SOC across continental alpine permafrost regions, including northern Eurasia and North America [24,55]. Accordingly, vertical SOM decomposition in northern permafrost peatlands may respond less drastically to climate warming than model projections. Nevertheless, divergent thermal sensitivity between continuous and patchy permafrost merits sustained attention. Collectively, our findings verify that mineral-associated protection suppresses the thermal sensitivity of organic carbon in peat wetlands of the Daxing’an Mountains permafrost area. When evaluating carbon–climate feedback of permafrost peat soils, models should incorporate the buffering capacity of mineral phases against SOC mineralization. Future work is needed to disentangle microbial regulatory effects on SOC mineralization and fully clarify how SOM stability shifts under thermal stimulation. At the same time, the constant-temperature cultivation in this study did not account for seasonal temperature fluctuations or freeze–thaw cycles; subsequent controlled field trials are required to further validate the mechanistic findings obtained in the laboratory.

## 5. Conclusions

Based on an 89-day incubation experiment and stable isotope analysis, this study quantified the stability response of SOM to experimental warming in permafrost peatlands at different stages of degradation. In isolated patchy permafrost (JGDQ), the cumulative SOC mineralisation was significantly higher than in continuous permafrost (TQ), ranging from 1719–2346 mg C kg^−1^ OC^−1^ at 5 °C and 3008–12,500 mg C kg^−1^ OC^−1^ at 15 °C it was 3008–8753 mg C kg^−1^ OC^−1^, whereas in the TQ area these figures were only 227–538 and 320–1250 mg C kg^−1^ OC^−1^, respectively. *Q*_10_ decreased with increasing soil depth, falling from 4.15 at the surface to 1.14 at depth, and remained consistently higher in JGDQ than in TQ. Mineral-rich deep soils (40–50 cm) exhibited a much weaker response to warming in terms of decomposition, with cumulative mineralisation increasing by only 1.41 to 1.75-fold, whereas organic-rich surface soils (0–10 cm) showed an increase of 2.32 to 3.73-fold, this confirms that physical protection mediated by Fe/Al oxides effectively inhibits the decomposition of SOM. In continuous permafrost, warming preferentially degraded the readily degradable fraction (rA2926), whereas in patchy permafrost, the stable fraction (rA1637) became the primary substrate for decomposition, indicating divergent stability trajectories of soil organic matter between the two permafrost types. Overall, mineral protection significantly reduced the temperature sensitivity of soil organic carbon mineralisation and delayed the transition of peatlands from carbon sinks to carbon sources under warming conditions. Future research should integrate microbial regulatory mechanisms to refine predictive models of permafrost carbon–climate feedback.

## Figures and Tables

**Table 1:** Physicochemical properties of soil at 0–50 cm depth in continuous and isolated patches permafrost (mean ± standard error, *n* = 3).

	Depth (cm)	SOC (g/kg)	TN (mg/g)	C:N Ratio	Clay wt%	Silt wt%	Sand wt%	δ ^13^C‰
TQ	0–10	88.17 ± 0.42	9.96 ± 0.13	8.98 ± 0.13	0.45 ± 0.02	40.86 ± 1.12	58.68 ± 0.98	−27.98 ± 0.10
	10–20	52.42 ± 3.59	5.82 ± 0.15	9.12 ± 0.32	5.37 ± 0.04	55.16 ± 1.55	39.45 ± 1.02	−27.91 ± 0.36
	20–30	37.60 ± 2.96	3.15 ± 0.18	11.89 ± 2.01	8.79 ± 0.49	48.94 ± 1.38	42.26± 1.87	−27.49 ± 0.20
	30–40	36.88 ± 6.23	2.81 ± 0.03	13.54 ± 1.54	9.06 ± 0.09	55.59 ± 1.22	35.35 ± 1.68	−27.29 ± 0.44
	40–50	26.48 ± 2.59	1.97 ± 0.12	13.67 ± 0.88	10.29 ± 0.91	49.41 ± 0.88	40.29 ± 1.42	−27.15 ± 0.21
JGDQ	0–10	348.40 ± 8.51	42.43 ± 0.23	8.36 ± 1.21	0.15 ± 0.01	21.30 ± 0.46	78.54 ± 0.41	−27.94 ± 0.05
	10–20	249.67 ± 2.76	28.81 ± 0.04	8.76 ± 0.89	0.74 ± 0.04	24.46 ± 0.23	74.79 ± 0.26	−27.84 ± 0.03
	20–30	86.09 ± 1.67	8.68 ± 0.05	9.32 ± 1.05	6.84 ± 0.35	55.99± 0.44	37.03± 0.46	−27.66 ± 0.08
	30–40	64.34 ± 0.12	5.96 ± 0.02	10.88 ± 2.98	3.47 ± 0.13	46.46 ± 0.71	50.06 ± 0.50	−27.68 ± 0.07
	40–50	55.12 ± 0.98	5.48 ± 0.05	11.01 ± 1.48	7.50 ± 0.26	51.68 ± 0.35	40.88 ± 1.48	−27.50 ± 0.01

Notes: SOC denotes soil organic carbon; TN denotes total nitrogen; C:N denotes the ratio of SOC to TN; Clay denotes particles < 2 μm; Silt denotes particles 2–20 μm; Sand denotes particles 20–200 μm; δ ^13^C denotes ^13^C isotopic abundance.

**Table 2:** Concentrations of Fe, Al and their oxides in the 0–50 cm soil layer of continuous and isolated patches permafrost (mean ± standard error, *n* = 3).

	Depth (cm)	Fe (mg/kg)	Fe_d_ (mg/kg)	Fe_o_ (mg/kg)	Fe_p_ (mg/kg)	Al (mg/kg)	Al_d_ (mg/kg)	Al_o_ (mg/kg)	Al_p_ (mg/kg)
TQ	0–10	41.07 ± 0.65	5.08 ± 0.51	5.02 ± 0.08	1.78 ± 0.22	25.78 ± 1.77	1.73 ± 0.04	0.89 ± 0.02	0.45 ± 0.04
	10–20	41.74 ± 1.15	5.65 ± 0.63	4.18 ± 0.16	1.58 ± 0.13	29.70 ± 8.40	1.40 ± 0.07	0.82 ± 0.06	0.29 ± 0.02
	20–30	42.94 ± 0.95	6.35 ± 0.52	3.46 ± 0.06	1.36 ± 0.23	35.77 ± 1.06	1.51 ± 0.07	0.75 ± 0.06	0.24 ± 0.03
	30–40	44.22 ± 0.73	6.47 ± 0.65	4.14 ± 0.25	1.29 ± 0.23	37.46 ± 3.68	1.60 ± 0.20	0.77 ± 0.02	0.25 ± 0.04
	40–50	46.16 ± 1.13	7.80 ± 0.36	3.93 ± 0.14	1.32 ± 0.09	39.56 ± 5.46	1.58 ± 0.11	0.75 ± 0.10	0.26 ± 0.02
JGDQ	0–10	16.84 ± 0.41	4.67 ± 0.13	3.79 ± 0.17	1.91 ± 0.58	23.56 ± 1.54	2.84 ± 0.11	2.23 ± 0.56	2.08 ± 0.55
	10–20	25.06 ± 0.31	5.66 ± 0.18	4.12 ± 0.09	1.44 ± 0.38	23.57 ± 1.65	3.55 ± 0.11	2.42 ± 0.35	1.28 ± 0.38
	20–30	36.22 ± 1.31	6.12 ± 0.34	2.39 ± 0.06	1.17 ± 0.04	28.51 ± 3.42	6.42 ± 0.19	1.41 ± 0.22	0.63 ± 0.02
	30–40	37.68 ± 0.94	6.54 ± 0.36	2.48 ± 0.08	1.02 ± 0.28	32.11 ± 3.16	6.15 ± 0.13	1.46 ± 0.06	0.47 ± 0.12
	40–50	39.42 ± 0.13	6.28 ± 0.17	2.62 ± 0.11	1.41 ± 0.10	35.46 ± 4.46	5.86 ± 0.05	1.54 ± 0.07	0.48 ± 0.03

Notes: Fe_d_/Al_d_ denotes free Fe and Al oxides; Fe_o_/Al_o_ denotes amorphous Fe and Al oxides; Fe_p_/Al_p_ denotes organically complexed Fe and Al oxides.

**Table 3:** A Spearman correlation analysis of the total organic carbon and soil δ ^13^C values with the different forms of the Fe and Al oxide contents (* *p* < 0.05; ** *p* < 0.01).

Indicators	δ ^13^C	Fe_d_ + Al_d_	Fe_o_ + Al_o_	Fe_p_ + Al_p_
SOC	−0.93 **	−0.89 **	0.39	0.75 *
δ ^13^C		0.04	−0.60	−0.35

Notes: Fe_d_ + Al_d_, Fe_o_ + Al_o_, Fe_p_ + Al_p_ denotes the sum of Fe_d_ and Al_d_, Fe_o_ and Al_o_, and Fe_p_ and Al_p_ in the soil layer 0–50 cm below the surface.

**Table 4:** The F-values of a two-way analysis of variance on the effects of fraction and temperature on the organic carbon features (* *p* < 0.05. ** *p* < 0.01).

	Cumulative Mineralization		δ ^13^C		rA1637		rA2926	
	*F*	*p*	*F*	*p*	*F*	*p*	*F*	*p*
permafrost type	27.29	0.00 **	34.36	0.00 **	11.85	0.00 **	7.27	0.02 *
temperature	9.38	0.00 **	4.63	0.04 *	0.18	0.68	0.52	0.48
permafrost type × temperature	6.25	0.02 *	5.35	0.03 *	0.06	0.81	0.15	0.70

Notes: rA1367 represents the relative peak area of stable organic matter in SOM, whilst rA2926 represents the relative peak area of labile organic matter in SOM.

## Data Availability

The raw data supporting the conclusions of this article will be made available by the authors on request.

## Supplementary Materials

The following supporting information can be downloaded at: https://www.mdpi.com/article/10.3390/molecules31173069/s1, Figure S1. Sampling sites in permafrost zones of Northeast China. Figure S2. Variation characteristics of soil microbial carbon content with depth in peatlands in different types of permafrost zones. Figure S3. FT-IR of DOM in the 0–10 cm and 40–50 cm soil layers in the continuous permafrost zone (TQ) and isolated patches permafrost zone (JGDQ) at different temperatures after 89 days of mineralization culture FT-IR spectrum ((a) TQ 0–10 cm; (b) TQ 40–50 cm (c) JGDQ 0–10 cm; (d) JGDQ 40–50 cm). Figure S4. Shows a revised analysis of organic carbon mineralization and the temperature sensitivity index (*Q*_10_) using various soil parameters. The size of the circle indicates the size of the correlation coefficient. The darker the circle, the stronger the correlation. Red indicates positive correlation, blue indicates negative correlation, and numbers in different colors indicate correlation coefficients. (Al_d_ + Fe_d_)/OC is the ratio of free Fe and Al oxides to organic carbon; (Al_o_ + Fe_o_)/OC is the ratio of poorly crystallized Fe and Al oxygen hydroxides to organic carbon; (Al_p_ + Fe_p_)/OC is the ratio of organic complex Fe and Al oxides to organic carbon content). Figure S5. Morphology of soil organic matter in the continuous permafrost zone (TQ) and isolated patches permafrost zone (JGDQ) at different temperatures and depths after 89 days of cultivation ((a) TQ-5 °C 0–10 cm; (b) TQ-15 °C 0–10 cm; (c) TQ-5 °C 40–50 cm; (d) TQ-15 °C 40–50 cm; (e) JGDQ-5 °C 0–10 cm; (f) JGDQ-15 °C 0–10 cm; (g) JGDQ-5 °C 40–50 cm; (h) JGDQ-15 °C 40–50 cm). Table S1. Sampling point location information and active layer thickness. Table S2 Physicochemical properties of soil at 0–50 cm depth in continuous and isolated patches permafrost (mean ± standard error, n = 3). Table S3 Concentrations of Fe, Al and their oxides in the 0–50 cm soil layer of continuous and isolated patches permafrost (mean ± standard error, *n* = 3).
