# Supplementary material for: The Degree of Permafrost Degradation Determines the Stability of Soil Organic Matter
Source: Molecules. 2026 Aug 31;31(17):3069. doi: 10.3390/molecules31173069 (PMC13567304; doi:10.3390/molecules31173069)
Supplement: Supplementary file 1 [file molecules-31-03069-s001.zip › molecules-4501338-supplementary.pdf]

Supplementary Materials

1.Materials and methods

1.1. Study Site

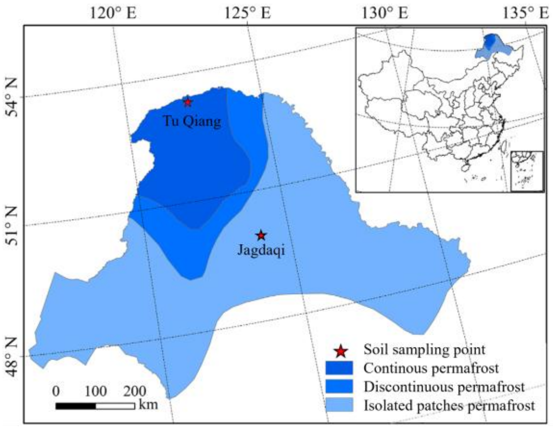

Figure S1. Sampling sites in permafrost zones of Northeast China

The above shows our three sampling sites: Tuqiang, Huzhong and Jiaogedaqi. In October 2023, we selected three parallel sampling plots in each area, spaced approximately 20 metres apart. At each sampling point, five soil samples were collected within a 1 metre × 1 metre sampling plot (four from the upper part of the plot and one from the centre), and these samples were subsequently combined into a single sample. At each sampling point, four samples (all taken from the same soil profile) were collected and placed in an incubator.

Table S1 Sampling point location information and active layer thickness

Location	Coordinates	Active layer thickness	Permafrost zone
Tu Qiang (TQ)	122°46′25"<br>52°55′01"	50±6 cm	continuous permafrost
Jiagedaqi (JGDQ)	124°13′21"<br>50°28′07"	80±5 cm	isolated patches permafrost

1.2. Sample processing method

Soil organic carbon (SOC) determination: Weigh 0.1 g of air-dried soil sample that has been ground and sieved, place it in a blank porcelain boat, slowly add 2–3 drops of 2 mol L^{-1} hydrochloric acid solution to remove inorganic carbon, then place the boat in an oven and dry to constant weight. Determine and analyze by high-temperature combustion method using a Multi N/C 3100 carbon-nitrogen analyzer (Jena, Germany).

Soil dissolved organic carbon (DOC) determination: Weigh fresh soil equivalent to 10 g dry weight into a 50 mL centrifuge tube, with a solid/liquid ratio of 1/6 (w/v). Shake at room temperature for 30 min, then centrifuge at high speed for 20 min. Filter the supernatant through a 0.45 μm membrane filter, and measure using a Multi N/C 3100 TOC analyzer (Jena, Germany). Calculate the dissolved organic carbon concentration based on the total organic carbon concentration in the extract.

Soil total nitrogen (TN) determination: Weigh 1.0000 g of soil sample that has been ground and sieved. For sandy soil samples, the amount can be adjusted appropriately according to actual conditions. During sample processing, an appropriate amount of acid should be added based on the specific properties of the soil to facilitate the subsequent digestion reaction. After digestion, use a SKALAR San++ continuous flow analyzer produced by Skalar (Netherlands) to quantitatively determine soil total nitrogen.

Stable isotope determination: Air-dried soil is passed through a 100-mesh sieve, then

acidified with 2 mol L^{-1} hydrochloric acid. During acidification, stir once every hour for a total of 6 hours. After acidification, wash the sample to neutrality, place it in an oven at 60 °C and dry for 24 hours. Calculate the corresponding sample mass based on the total organic carbon value.

The total amounts of Fe/Al, free oxides (Fe_d, Al_d), poorly crystalline oxyhydroxides (Fe_o, Al_o), and organically complexed oxides (Fe_p, Al_p) were determined using selective extraction methods [34]. The Fed and Al_d in the soil samples were extracted using 0.3 mol L^{-1} of sodium citrate solution and 1 mol L^{-1} of sodium bicarbonate solution. Then, the samples were heated to 80 °C in a water bath, after that added sodium dithionite, they were constantly stirred and maintained for 15 min. After cooling, centrifugation, and separation, the residue was washed in the centrifuge tube 2–3 times with 1 mol L^{-1} NaCl. Fe_o and Al_o were extracted using ammonium oxalate buffer at 1:50, and they were shaken for 2 h at room temperature. The Fe_p and Al_p were extracted using sodium pyrophosphate solution at the solid/liquid ratio of 1:20, and they were shaken for 2 h. After centrifugation, the supernatants were measured using a sequential plasma spectrometer (ICPS-7500, Shimadzu, Japan).

In this study, two levels of warming experiments were set up at 15 °C and 5 °C, with the incubation moisture maintained at the in-situ moisture condition, without any special treatment. Soil samples (10 g dry weight) passed through a 2 mm mesh sieve were placed in 500 mL sealed glass bottles with rubber stoppers, and incubated in incubators (Thermo Fisher Scientific, USA) set at the specified temperatures for a certain period.Three empty bottles without soil were placed in each incubator as blank controls. A 10-day pre-incubation was conducted prior to the formal culture to balance the effects of sample transport and homogenization on the microorganisms, allowing them to regain activity [44]. Before collecting gas samples, connect the gas collection syringe to the three-way valve on the rubber stopper of the culture flask and collect 15 mL of gas. After collecting the gas sample, flush the culture flask with air for approximately 30 minutes to maintain the initial gas concentration. When not collecting gas, open the three-way valve to supply oxygen to the sample. Weigh the flask weekly and add an appropriate amount of deionised water to compensate for any reduction in soil moisture content.

$$F = \rho \times \Delta C \times V \times \frac{273}{(273+T)\times W}$$

$$C = \sum_{i=n}^{n} \frac{F_i + F_{i+1}}{2\times(t_{i+1}-t_i)\times 24}$$

$$Q_{10} = \left(\frac{K_1}{K_2}\right)^{\frac{10}{T_2-T_1}}$$

In the equation, F represents the rate of soil organic carbon mineralisation, expressed in mg kg^{-1} h^{-1}; ρ represents the concentration of CO_2 under standard conditions, expressed in g L^{-1}; ΔC represents the difference in CO_2 concentration per unit time following incubation under sealed conditions; V represents the volume of the culture flask, expressed in mL; T represents the set incubation temperature, expressed in °C; and W represents the dry weight of the culture soil, expressed in kg. In this equation, C represents the cumulative mineralisation of soil organic carbon, in mg kg^{-1}; F represents the rate of soil organic carbon mineralisation, in mg kg^{-1} h^{-1}; i represents the measurement number; t_{i+1}-t_i represents the time interval between two consecutive measurements; and n represents the total number of measurements within the incubation cycle. The temperature sensitivity of soil organic carbon mineralisation refers to the multiple by which the soil organic carbon mineralisation rate changes following a 10 °C change in temperature, and is generally denoted by Q_{10}. In this equation, T_1 is set to 5 °C and T_2 to 15 °C; K_1 and K_2 represent the soil organic carbon mineralisation rates at 5 °C and 15 °C, respectively.

Microbial carbon (MBC) determination: Based on the chloroform fumigation–K_2SO_4 extraction method, 10 g of soil sample was weighed and fumigated with chloroform for 24 hours in the dark; both fumigated and unfumigated samples were extracted with 0.5 mol L^{-1} K_2SO_4 for approximately 30 minutes, with a soil/liquid ratio of 1:4. The organic carbon concentration in the extract was determined using a Multi N/C 3100 TOC analyser (Jena, Germany).

The carbon and nitrogen content of the microbial biomass is calculated using the following formula:

$$MBC = \frac{E_C}{0.45}$$

In the equation, MBC represents microbial biomass carbon (mg kg^{-1}), EC refers to the difference in organic carbon between the extracts of fumigated and unfumigated samples, and 0.45 is the calculation factor.

2. Results and discussion

Table S2 Physicochemical properties of soil at 0-50 cm depth in continuous and isolated patches permafrost (mean ± standard error, n=3).

		SOC (g/kg)				TN (mg/g)				C:N ratio				Clay (wt%)				Silt (wt%)				Sand (wt%)				δ13C (‰)			
	Depth	Mean	SE	SD	CV	Mean	SE	SD	CV	Mean	SE	SD	CV	Mean	SE	SD	CV	Mean	SE	SD	CV	Mean	SE	SD	CV	Mean	SE	SD	CV
TQ	0–10	88.17	0.42	0.73	0.83	9.96	0.13	0.23	2.26	8.98	0.13	0.23	2.51	0.45	0.02	0.03	7.70	40.86	1.12	1.94	4.75	58.68	0.98	1.70	2.89	-27.98	0.10	0.17	0.62
	10–20	52.42	3.59	6.22	11.86	5.82	0.15	0.26	4.46	9.12	0.32	0.55	6.08	5.37	0.04	0.07	1.29	55.16	1.55	2.68	4.87	39.45	1.02	1.77	4.48	-27.91	0.36	0.62	2.23
	20–30	37.60	2.96	5.13	13.64	3.15	0.18	0.31	9.90	11.89	2.01	3.48	29.28	8.79	0.49	0.85	9.66	48.94	1.38	2.39	4.88	42.26	1.87	3.24	7.66	-27.49	0.20	0.35	1.26
	30–40	36.88	6.23	10.79	29.26	2.81	0.03	0.05	1.85	13.54	1.54	2.67	19.70	9.06	0.09	0.16	1.72	55.59	1.22	2.11	3.80	35.35	1.68	2.91	8.23	-27.29	0.44	0.76	2.79
	40–50	26.48	2.59	4.49	16.94	1.97	0.12	0.21	10.55	13.67	0.88	1.52	11.15	10.29	0.91	1.58	15.32	49.41	0.88	1.52	3.08	40.29	1.42	2.46	6.10	-27.15	0.21	0.36	1.34
JGDQ	0–10	348.40	8.51	14.74	4.23	42.43	0.23	0.40	0.94	8.36	1.21	2.10	25.07	0.15	0.01	0.02	11.55	21.30	0.46	0.80	3.74	78.54	0.41	0.71	0.90	-27.94	0.05	0.09	0.31
	10–20	249.67	2.76	4.78	1.91	28.81	0.04	0.07	0.24	8.76	0.89	1.54	17.60	0.74	0.04	0.07	9.36	24.46	0.23	0.40	1.63	74.79	0.26	0.45	0.60	-27.84	0.03	0.05	0.19
	20–30	86.09	1.67	2.89	3.36	8.68	0.05	0.09	1.00	9.32	1.05	1.82	19.51	6.84	0.35	0.61	8.86	55.99	0.44	0.76	1.36	37.03	0.46	0.80	2.15	-27.66	0.08	0.14	0.50
	30–40	64.34	0.12	0.21	0.32	5.96	0.02	0.03	0.58	10.88	2.98	5.16	47.44	3.47	0.13	0.23	6.49	46.46	0.71	1.23	2.65	50.06	0.50	0.87	1.73	-27.68	0.07	0.12	0.44
	40–50	55.12	0.98	1.70	3.08	5.48	0.05	0.09	1.58	11.01	1.48	2.56	23.28	7.50	0.26	0.45	6.00	51.68	0.35	0.61	1.17	40.88	1.48	2.56	6.27	-27.50	0.01	0.02	0.06

Table S3 Concentrations of Fe, Al and their oxides in the 0-50 cm soil layer of continuous and isolated patches permafrost (mean ± standard error, *n*=3).

	Depth (cm)	Fe (mg/kg)				Fe_d (mg/kg)				Fe_o (mg/kg)				Fe_p (mg/kg)				Al (mg/kg)				Al_d (mg/kg)				Al_o (mg/kg)				Al_p (mg/kg)			
		Mean	SE	SD	CV	Mean	SE	SD	CV	Mean	SE	SD	CV	Mean	SE	SD	CV	Mean	SE	SD	CV	Mean	SE	SD	CV	Mean	SE	SD	CV	Mean	SE	SD	CV
TQ	0–10	41.07	0.65	1.13	2.74	5.08	0.51	0.88	17.39	5.02	0.08	0.14	2.76	1.78	0.22	0.38	21.41	25.78	1.77	3.07	11.89	1.73	0.04	0.07	4.00	0.89	0.02	0.03	3.89	0.45	0.04	0.07	15.40
	10–20	41.74	1.15	1.99	4.77	5.65	0.63	1.09	19.31	4.18	0.16	0.28	6.63	1.58	0.13	0.23	14.25	29.70	8.40	14.55	28.99	1.40	0.07	0.12	8.66	0.82	0.06	0.10	12.67	0.29	0.02	0.03	11.95
	20–30	42.94	0.95	1.65	3.83	6.35	0.52	0.90	14.18	3.46	0.06	0.10	3.00	1.36	0.23	0.40	29.29	35.77	1.06	1.84	5.13	1.51	0.07	0.12	8.03	0.75	0.06	0.10	13.86	0.24	0.03	0.05	21.65
	30–40	44.22	0.73	1.26	2.86	6.47	0.65	1.13	17.40	4.14	0.25	0.43	10.46	1.29	0.23	0.40	30.88	37.46	3.68	6.37	17.02	1.60	0.20	0.35	21.65	0.77	0.02	0.03	4.50	0.25	0.04	0.07	27.71
	40–50	46.16	1.13	1.96	4.24	7.80	0.36	0.62	7.99	3.93	0.14	0.24	6.17	1.32	0.09	0.16	11.81	39.56	5.46	9.46	23.91	1.58	0.11	0.19	12.06	0.75	0.10	0.17	23.09	0.26	0.02	0.03	13.32
JGDQ	0–10	16.84	0.41	0.71	4.22	4.67	0.13	0.23	4.82	3.79	0.17	0.29	7.77	1.91	0.58	1.00	32.60	23.56	1.54	2.67	11.32	2.84	0.11	0.19	6.71	2.23	0.56	0.97	23.50	2.08	0.55	0.95	25.80
	10–20	25.06	0.31	0.54	2.14	5.66	0.18	0.31	5.51	4.12	0.09	0.16	3.78	1.44	0.38	0.66	25.71	23.57	1.65	2.86	12.13	3.55	0.11	0.19	5.37	2.42	0.35	0.61	25.05	1.28	0.38	0.66	21.42
	20–30	36.22	1.31	2.27	6.26	6.12	0.34	0.59	9.62	2.39	0.06	0.10	4.35	1.17	0.04	0.07	5.92	28.51	3.42	5.92	20.78	6.42	0.19	0.33	5.13	1.41	0.22	0.38	27.02	0.63	0.02	0.03	5.50
	30–40	37.68	0.94	1.63	4.32	6.54	0.36	0.62	9.53	2.48	0.08	0.14	5.59	1.02	0.28	0.48	47.55	32.11	3.16	5.47	17.05	6.15	0.13	0.23	3.66	1.46	0.06	0.10	7.12	0.47	0.12	0.21	24.22
	40–50	39.42	0.13	0.23	0.57	6.28	0.17	0.29	4.69	2.62	0.11	0.19	7.27	1.41	0.10	0.17	12.28	35.46	4.46	7.72	21.78	5.86	0.05	0.09	1.48	1.54	0.07	0.12	7.87	0.48	0.03	0.05	10.83

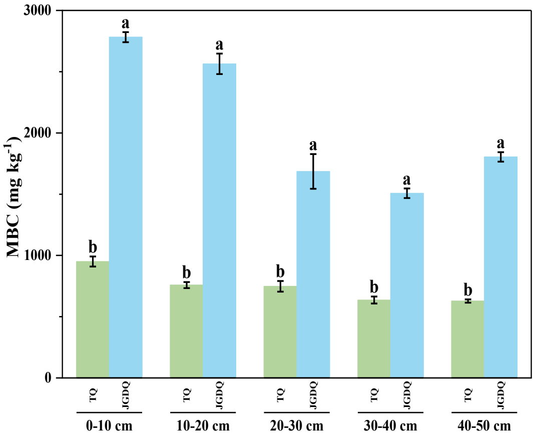

Figure S2. Variation characteristics of soil microbial carbon content with depth in peatlands in different types of permafrost zones.

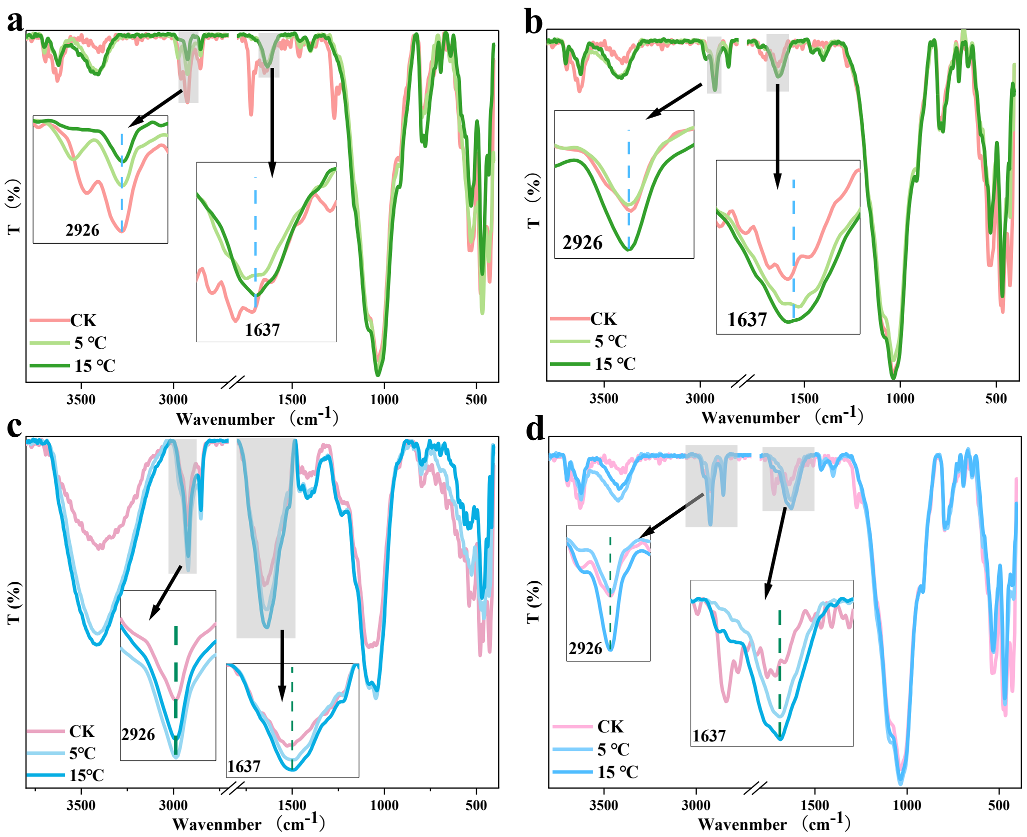

Figure S3. FT-IR of DOM in the 0-10 cm and 40-50 cm soil layers in the continuous permafrost zone (TQ) and isolated patches permafrost zone (JGDQ) at different temperatures after 89 days of mineralization culture FT- IR spectrum (a: TQ 0-10 cm; b: TQ 40-50 cm c: JGDQ 0-10 cm; d: JGDQ 40-50 cm).

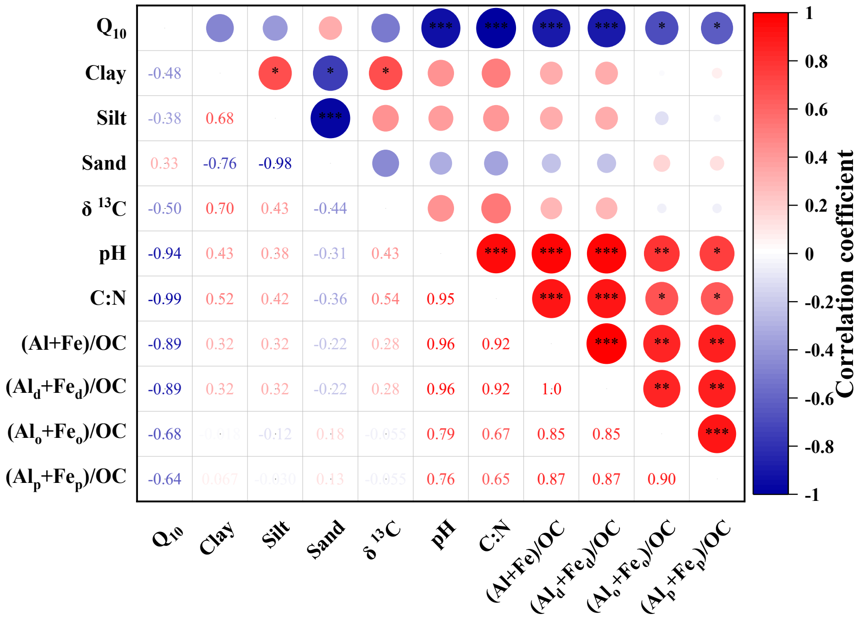

Figure S4. Shows a revised analysis of organic carbon mineralization and temperature sensitivity index (Q_{10}) using various soil parameters. The size of the circle indicates the size of the correlation coefficient. The darker the circle, the stronger the correlation. Red indicates positive correlation, blue indicates negative correlation, and numbers in different colors indicate correlation coefficients. ((Al_d+Fe_d)/OC is the ratio of free Fe and Al oxides to organic carbon; (Al_o+Fe_o)/OC is the ratio of poorly crystallized Fe and Al oxygen hydroxides to organic carbon; (Al_p+Fe_p)/OC is the ratio of organic complex Fe and Al oxides to organic carbon content).

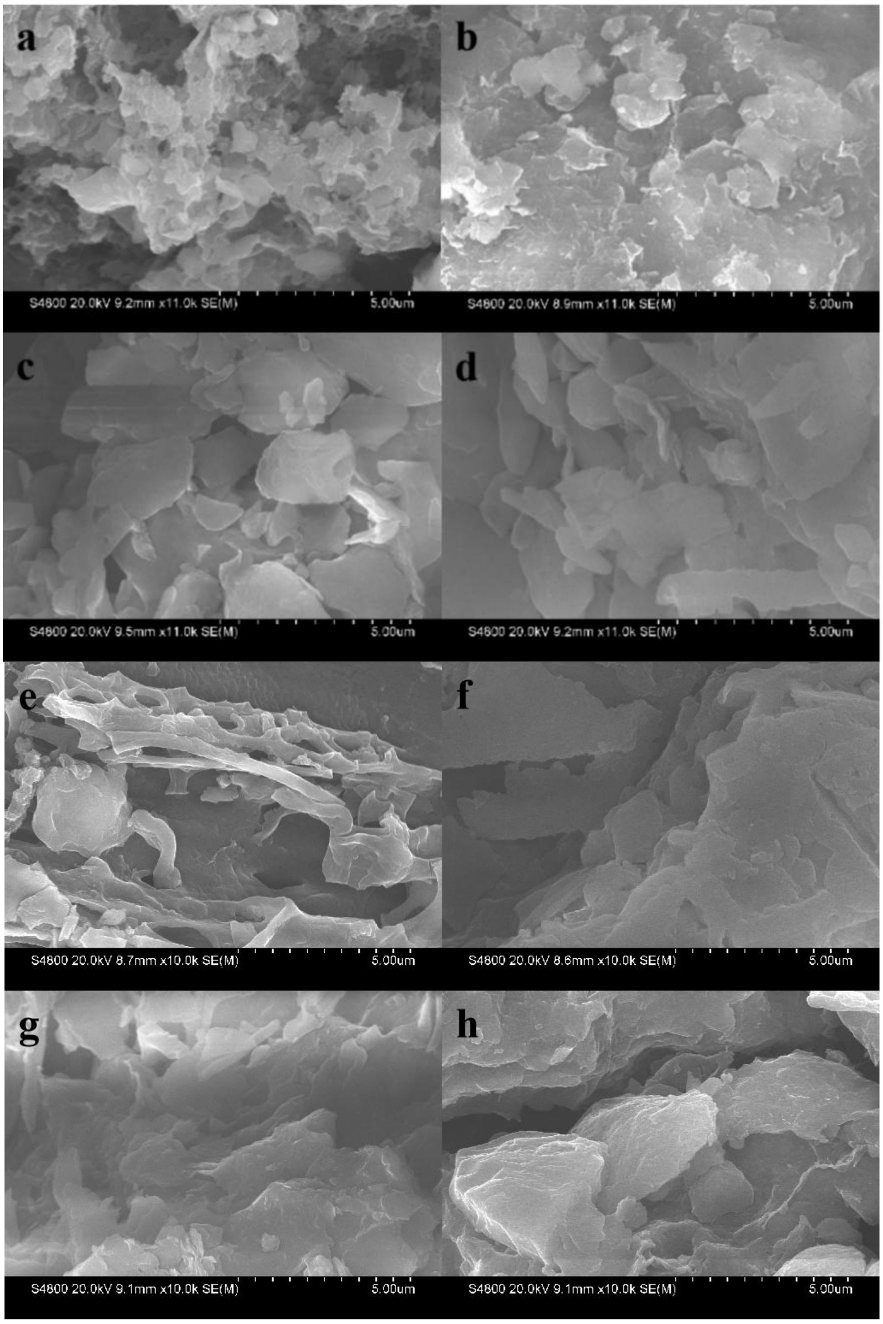

Figure S5. Morphology of soil organic matter in continuous permafrost zone (TQ) and isolated patches permafrost zone (JGDQ) at different temperatures and depths after 89 days of cultivation (a: TQ-5 °C 0-10 cm; b: TQ-15 °C 0-10 cm; c: TQ-5 °C 40-50 cm; d: TQ-15 °C 40-50 cm; e: JGDQ - 5 °C 0-10 cm; f: JGDQ -15 °C 0-10 cm; g: JGDQ -5 °C 40-50 cm; h: JGDQ -15 °C 40-50 cm)
